# CT Imaging‐based radiomics predicts the pain relief of Strontium‐89 in treating tumor‐induced bone metastases

**DOI:** 10.1002/acm2.70189

**Published:** 2025-07-15

**Authors:** Danzhou Fang, Yaofeng Xiao, Shunhao Zhou, Feng Shi, Yuwei Xia, Gengbiao Yuan, Xiaojiao Xiang

**Affiliations:** ^1^ Department of Nuclear Medicine The Second Affiliated Hospital of Chongqing Medical University Chongqing China; ^2^ Shanghai United Imaging Intelligence Shanghai China

**Keywords:** bone relief, radiomics, Strontium‐89

## Abstract

**Background:**

Bone metastasis is a common complication in advanced malignancies, often resulting in severe pain and reduced quality of life. Radiopharmaceuticals like Strontium‐89 (^89^Sr) are commonly used for palliative treatment to alleviate bone pain associated with metastases. This study explores the potential of radiomics analysis in predicting the effectiveness of ^89^Sr treatment for pain relief in patients with bone metastases.

**Methods:**

The study analyzed clinical and imaging data from 146 patients with bone metastases, specifically focusing on two types of lesions: osteolytic and osteoblastic. Pain relief was assessed by the step of the WHO pain ladder required for pain relief, along with a reduction in opioid dosage, indicating effective pain management. Based on exploratory analysis, a Bagging Decision Tree machine learning model was selected for outcome prediction in osteolytic lesions, while the XGBoost model was utilized for osteoblastic lesions. Both models leveraged radiomics features extracted from these lesions to improve predictive accuracy. Model performance was evaluated using the area under the receiver operating characteristic curve (AUC), along with sensitivity, specificity, accuracy, and calibration curves.

**Results:**

The pain relief rate for osteolytic metastases was 58.33%, and for osteoblastic metastases, it was 62.16%. The Bagging Decision Tree model achieved an AUC of 0.991 in the training set and 0.889 in the test set for osteolytic lesions. For osteoblastic lesions, the XGBoost model yielded robust results, with an AUC of 0.970 in the training set and 0.958 in the test set.

**Conclusion:**

This study shows promise in predicting pain relief outcomes of ^89^Sr treatment in patients with bone metastases.

**Abbreviations**
AUC: Area under the receiver operating characteristic curve
ROC: receiver operating characteristic curve
WHO: world health organization

## 1 INTRODUCTION

Bone metastasis is a common complication in advanced malignancies.^1^ One of its most debilitating manifestations is severe pain, which not only limits mobility and disrupts sleep but also significantly diminishes the patient's overall quality of life.^2^ Several therapeutic strategies are available to manage bone pain and metastasis, including surgery, external irradiation, chemotherapy, hormone therapy, and radionuclide therapy.^3^ Among these, radiopharmaceuticals have increasingly emerged as a promising option for alleviating metastatic bone pain.^4^ Strontium‐89 chloride (^89^Sr) is a notable bone‐seeking radiopharmaceutical, with properties that make it particularly suitable for internal radiation therapy. It emits pure β‐particles, which not only may contribute to tumor reduction but also help alleviate pressure on the periosteum and bone marrow cavity. In addition, these particles can interfere with the depolarization of nerve endings, thereby modulating pain signal transmission.^5^ ^89^Sr exhibits a natural affinity for metabolically active bone, similar to calcium, and predominantly accumulates in regions of increased osteoblastic activity—sites of new bone formation. At metastatic lesions with active osteoblasts, radiation uptake can be up to ten times that of normal bone tissue, enabling targeted radiation delivery that provides pain relief comparable to high‐dose, extended‐cycle external beam radiation therapy.^6^ However, osteolytic lesions, which are primarily characterized by bone resorption and minimal osteoblastic activity, show limited uptake of ^89^Sr. Nevertheless, ^89^Sr may still offer some pain relief in osteolytic metastases, as its radiative effects may target surrounding tissues or areas of reactive bone formation at the lesion margins.^7^

Radiomics, a method that enables the automated extraction of image features for data mining, has been successfully applied in nuclear medicine for prognosis prediction and therapeutic assessment. Studies have demonstrated its efficacy in various clinical scenarios, such as predicting treatment response to transarterial chemoembolization in hepatocellular carcinoma and assessing overall survival in oncologic patients based on PET/CT imaging.^8^ In nuclear medicine, radiomics has been utilized to extract quantitative imaging biomarkers from PET and SPECT scans, aiding in the differentiation of tumor subtypes, predicting disease progression, and optimizing personalized treatment strategies.^9^ Additionally, it has been instrumental in evaluating the predictive role of radiomics in anti‐PD‐1 therapy in patients with hepatic cell carcinoma.^10^ Currently, radiomics is widely utilized in oncological PET/CT analysis.^11^ However, its application in therapeutic nuclear medicine, particularly in the context of radionuclide therapy, remains limited.^12^ Few studies have explored how radiomic features derived from imaging can predict the therapeutic response to agents such as ^89^Sr or ^177^Lu.^13^ This gap limits our ability to assess therapy non‐invasively and personalize it for patients receiving radiopharmaceutical treatment.

The aim of this study is to develop and evaluate a CT‐based radiomics model to predict pain relief outcomes in patients with bone metastases receiving ^89^Sr radionuclide therapy. By identifying relevant radiomic features prior to treatment, we seek to provide a non‐invasive tool for predicting therapeutic efficacy, which could ultimately support more personalized decision‐making in therapeutic nuclear medicine.

## 2 MATERIAL AND METHODS

### 2.1 Patient

**FIGURE 1:**
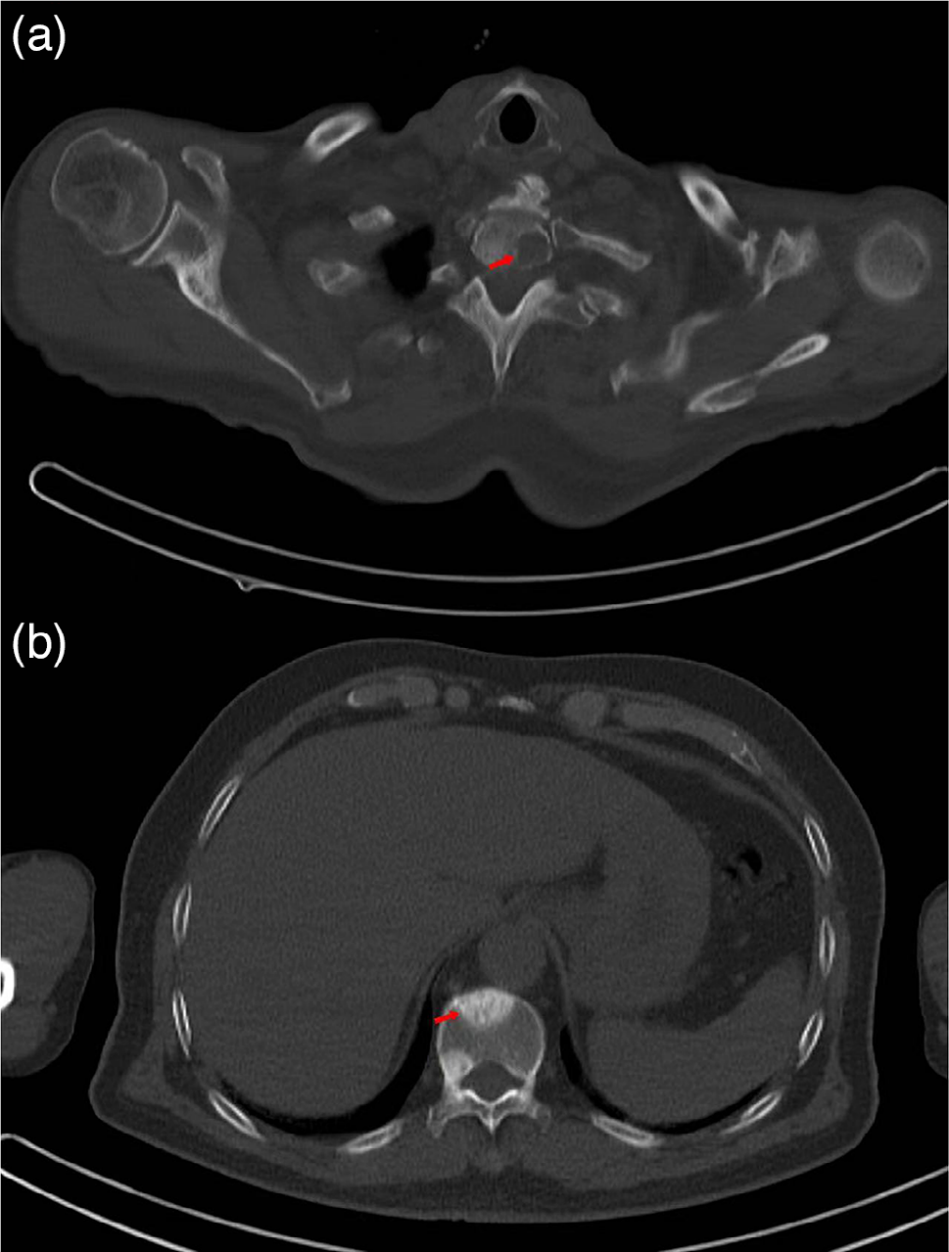
(a) Shows the characteristics of osteolytic bone destruction. (b) represents the characteristics of osteoblastic bone destruction.

This retrospective study received approval from the institutional review board of our hospital and was performed in accordance with the ethical standards laid down in the 1964 Declaration of Helsinki and all subsequent revisions. Due to the retrospective design, informed consent was waived. We screened patients older than 18 years with bone metastases who underwent CT scanning at our institution from February 2018 to July 2023, prior to receiving ^89^Sr therapy. CT images were presented in DICOM format and acquired using a Siemens spiral CT scanner. The parameters are as follows: sweep mode, spiral; tube voltage, 120 kV; tube current, 130 mAs; rotation time, 0.5s; and matrix size, 512 × 512. The reconstruction kernel used for all CT scans was the B50f kernel, and this kernel was consistently applied across all patients in the cohort to ensure uniformity and minimize variability in feature extraction. Inclusion Criteria: (1)CT scans revealing bone lesions, (2)Clinical symptoms of bone pain necessitating long‐term analgesic use, (3)Adequate bone marrow function as indicated by: Platelet count ≥ 100×10^9^/L, Leukocyte count ≥ 3.0×10^9^/L, Hemoglobin levels ≥ 6.0 mmol/L or 9.67 g/dL, (4)Serum creatinine≤180 µmol/L and glomerular filtration rate≥30 mL/min. Exclusion Criteria: (1)Previous treatment with external irradiation or other therapies for bone metastases, (2)Death within 3 months following ^89^Sr therapy, (3) Presence of fractures, spinal cord compression, or soft tissue tumors. (4)Diagnosis of multiple myeloma or primary bone neoplasms. Osteolytic lesions are defined as low‐density lesions in the bone cortex (as shown in Figure 1a), while osteoblastic lesions are defined as high‐density lesions surrounded by normal bone marrow or fused with the bone cortex (as shown in Figure 1b).^14^ We collated essential patient information, including age, sex, primary tumor type, liver and kidney function, blood routine test results, treatment methods, and disease progression.

### 2.2 Analgesic treatment

As the primary approach for managing bone pain, patients received analgesic treatment according to the World Health Organization (WHO) pain ladder. This standardized protocol escalates treatment based on pain severity, starting with nonsteroidal anti‐inflammatory drugs such as aspirin, ibuprofen, or naproxen, progressing to weak opioids like codeine or hydrocodone, and, if necessary, advancing to potent opioids such as morphine, hydromorphone, or fentanyl.^15^ In this study, the outcome variable was defined as whether pain relief occurred following ^89^Sr therapy. Analgesic use was evaluated based on shifts in the WHO pain ladder category before and after treatment. “Effective” indicates patients who experienced pain relief after ^89^Sr treatment, demonstrated by a downward shift in analgesic use according to the WHO pain ladder. “Ineffective” indicates patients who did not experience pain relief, showing no change or an upward shift in analgesic use. This approach provided a standardized and objective measure of ^89^Sr's impact on bone pain management.

### 2.3 Segmentations

A total of 146 patients were included in the present study, 93 males (63.70%) and 53 females (36.30%). There were 72 osteolytic lesions and 74 osteoblastic lesions. Subsequently, based on the type of lesion, the patients were each divided into two groups, with a ratio of 7:3 for each group. Specifically, approximately 51 patients with osteolytic lesions were assigned to the training set, with 21 to the test set; approximately 52 patients with osteoblastic lesions were allocated to the training set, with 22 allocated to the test set. All regions of interest (ROIs) were manually delineated around spinal bone metastases to ensure consistency in anatomical context and reduce variability from adjacent tissues. The segmentation was performed independently by two radiologists, each with over 5 years of experience, and subsequently reviewed by a senior radiologist with more than 15 years of clinical experience. In cases of discrepancy, the senior radiologist made minor edits to the delineations to ensure accuracy and uniformity.

### 2.4 Feature Selection

The image processing and feature extraction procedures were conducted in accordance with the guidelines of the Imaging Biomarker Standardization Initiative (IBSI).^16^ Feature standardization, selection, and model building were performed using the uAI Research Portal (Version: 20,220,230, http://urp.united‐imaging.com). Additional data processing and analysis were carried out using the “scikit‐learn” package (https://scikit‐learn.org) and “matplotlib” (https://matplotlib.org). Feature selection followed a systematic approach to identify the most relevant features for classifying osteolytic and osteoblastic lesions. The SelectKBest method, based on the chi‐square test, was applied to rank features by their statistical significance in distinguishing between the two groups. The choice of *k* = 8 was not arbitrary but rather determined through an iterative process, evaluating different values of k based on model performance metrics, including classification accuracy, the area under the receiver operating characteristic curve (AUC), and calibration measures. The aim was to retain a sufficient number of features that contributed meaningfully to the classification task while eliminating redundant or irrelevant features.

### 2.5 Osteolytic Lesions

Radiomics features were extracted from osteolytic lesions and categorized into first‐order statistics, shape‐based features, texture features, and high‐order features. For osteolytic image features, *Z*‐score normalization was applied to mitigate the dimensional effects of different features. Subsequently, the Select K Best method was applied to remove features that did not significantly differ between the two groups, with *K* = 8 determined based on exploratory analysis to balance model complexity and performance. Finally, the least absolute shrinkage and selection operator (LASSO) was utilized to select the features highly correlated with prognosis as the optimal radiomics features. The process of LASSO is shown in Figure S1.

### 2.6 Osteoblastic lesions

Radiomics features were extracted from osteoblastic lesions. For features of osteoblastic images, Z‐score normalization was employed to mitigate the dimensional effects of different features. Subsequently, the LASSO was utilized to select the features highly correlated with prognosis as the optimal radiomics features. After LASSO selection, 10 features were ultimately retained, as illustrated in Figure S2.

### 2.7 Model construction

For the classification of osteolytic and osteoblastic lesions, we employed different machine learning models optimized with specific parameters. Linear discriminant analysis was used as the dimensionality reduction method for both lesion types, retaining eight features for classification. The selection of different classifiers for osteolytic and osteoblastic lesions was based on empirical performance evaluation and the nature of the underlying lesion characteristics. Bagging Decision Tree was chosen for osteolytic lesions due to their robustness in handling complex decision boundaries and reducing variance through ensemble learning. In contrast, XGBoost was selected for osteoblastic lesions because of its ability to capture intricate feature interactions and its effectiveness in optimizing performance with structured data. Comparative experiments were conducted to assess different models, and the final selections were based on superior classification metrics observed during model validation.

For osteolytic lesions, a Bagging Decision Tree model was implemented with a maximum depth of 4, using all available features (max_features = 1.0) and samples (max_samples = 1.0) for training. A total of 100 decision tree estimators (n_estimators = 100) were aggregated, and a probability threshold of 0.5 was applied for classification.

For osteoblastic lesions, an XGBoost classifier with a binary logistic objective function was used. The model had a learning rate (eta) of 0.3, no regularization penalty (gamma = 0.0), and a maximum tree depth of 3, with 100 estimators (n_estimators = 100) trained for classification. A probability threshold of 0.5 was applied for final classification.

The hyperparameters for both models were selected based on typical values used in similar applications and practical considerations. For the Bagging Decision Tree, the maximum depth was set to 4, and 100 estimators were chosen based on standard practice. For XGBoost, a learning rate of 0.3 and a tree depth of 3 were used to balance model complexity and performance. Five‐fold cross‐validation was applied to both the training and validation cohorts.

### 2.8 Model performance evaluation

Model performance was then evaluated on the held‐out validation cohort. The receiver operating characteristic curve (ROC) was plotted, and performance metrics, including AUC, sensitivity, specificity, accuracy, and F1‐score, were calculated separately for both the training (via cross‐validation) and validation cohorts. Decision curve analysis was conducted to evaluate the clinical utility of the predictive model by quantifying the net benefits across a range of threshold probabilities. The “treat all” strategy assumes that all patients receive treatment regardless of model prediction, while the “treat none” strategy assumes that no patients are treated. These strategies were used as reference lines to compare the net benefit of the radiomics model.^17^

### 2.9 Statistical analysis

All statistical analyses were performed using SPSS software (Version 26.0). Continuous variables were expressed as means ± standard deviation. The normality of continuous data was tested using the Shapiro–Wilk test, and homogeneity of variances was assessed with Levene's test. As the data met the assumptions for parametric analysis, comparisons of continuous variables were conducted using independent‐samples *t*‐tests. Categorical variables were expressed as frequencies. Comparisons of categorical variables between prognosis groups were performed using the chi‐square test, as all expected cell counts were greater than 5. A *p*‐value less than 0.05 was considered statistically significant.

## 3 RESULTS

### 3.1 Basic characteristics

As shown in Table 1, there was no statistically significant difference in prognosis between the two types of bone lesions with regard to age and gender(*p* > 0.05). The primary tumors of osteoblastic lesions included 51 lung cancers (68.92%), 8 liver cancers (10.81%), 5 breast cancers (6.76%), 3 prostate cancers (4.05%), and other types of tumors. The primary tumors of osteolytic lesions included 44 lung cancers (61.11%), 8 prostate cancers (11.11%), 7 liver cancers (9.72%), 4 breast cancers (5.56%), and other types of tumors.

**TABLE 1:** The basic characteristics of patients with osteolytic and osteoblastic lesions.

Type	Osteolytic			Osteoblastic		
Prognosis	Ineffective^a^	Effective^b^	*p*	Ineffective	Effective	*p*
Sex(M/F)	11/19	23/19	0.467	19/9	32/14	0.878
Age(years)	62.67±10.784	64.62±10.546	0.446	65.11±11.060	63.41±12.277	0.552

^a^ “Ineffective” indicates patients who did not experience pain relief, showing no change or an upward shift in

analgesic use.

^b^ “Effective” indicates patients who experienced pain relief after 89Sr treatment, demonstrated by a

downward shift in analgesic use according to the WHO pain ladder.

### 3.2 Model performance

**FIGURE 2:**
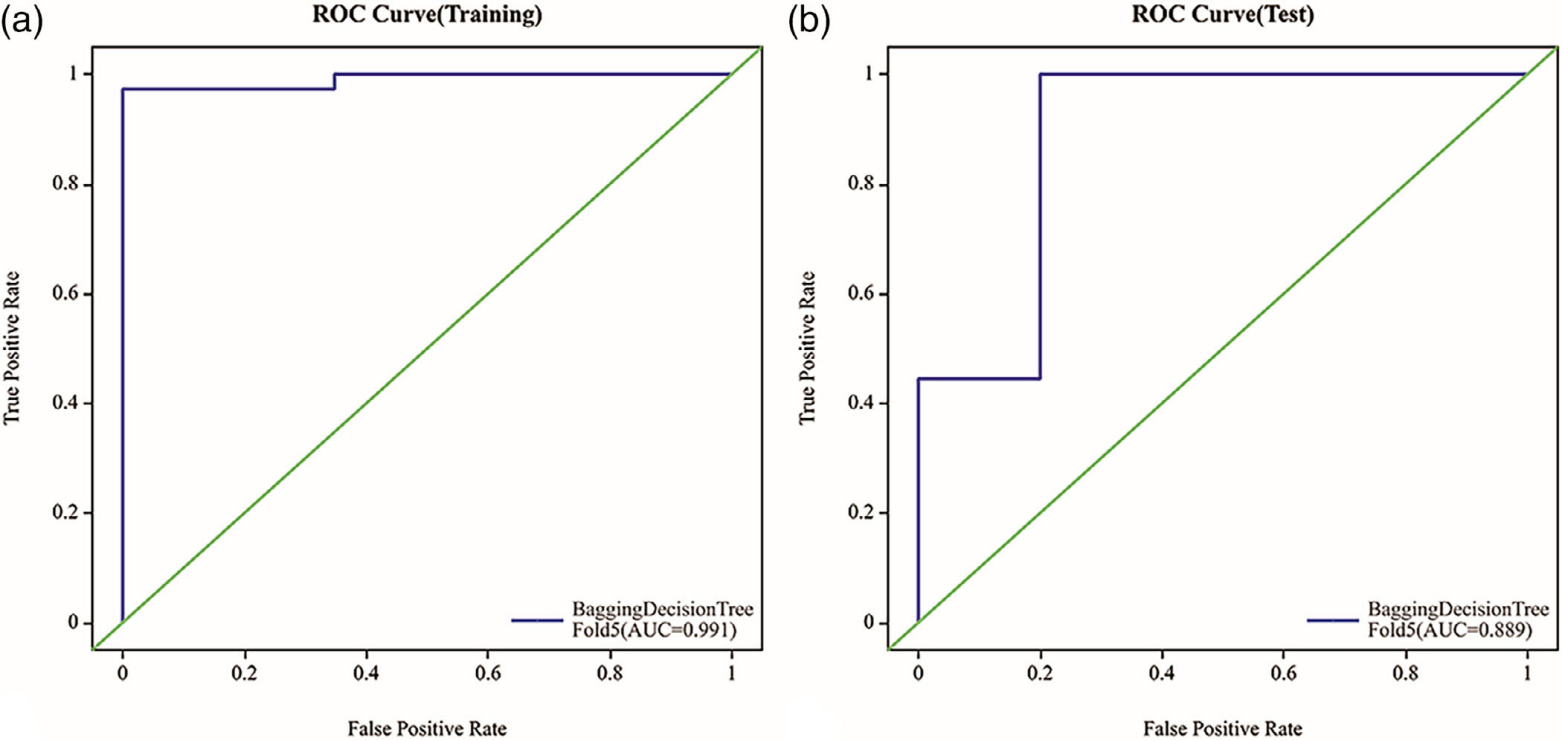
The AUC of the Bagging Decision Tree model. The AUC of the training set is 0.991, and the AUC of the validation set is 0.889.

**FIGURE 3:**
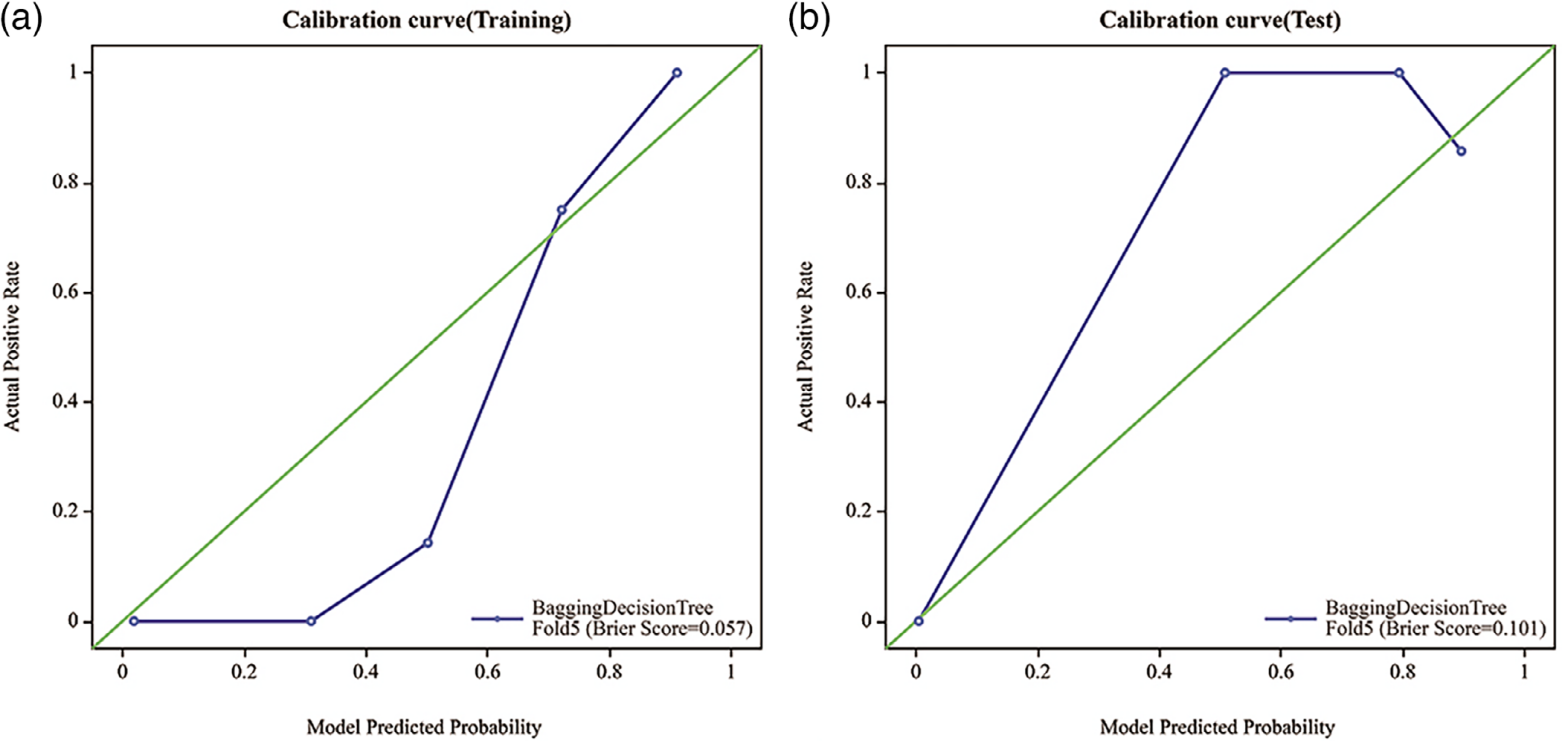
The calibration curve of the Bagging Decision Tree model.

**FIGURE 4:**
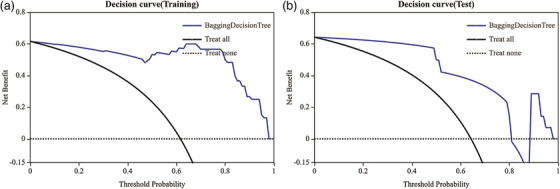
The decision curve analysis of the Bagging Decision Tree model.

In the training set, the Bagging Decision Tree model for osteolytic lesions demonstrated high predictive accuracy, achieving an AUC of 0.991 (95% CI: 0.971–1), with a sensitivity of 0.973 and a specificity of 0.826. However, its performance on the test set was lower, with an AUC of 0.889 (95% CI 0.66‐1), along with a sensitivity of 0.889 and a specificity of 0.8 (as shown in Figure 2). In the training data, the calibration curve closely matched the ideal diagonal line (represented by the green line) until predicted probabilities reached around 0.8. Beyond this point, the model started to deviate, suggesting a possible overestimation of positive outcomes at high probability values. The Brier Score of 0.057 underscored the model's strong calibration in the training set, as a lower Brier Score indicates better calibration. However, when examining the test data, the calibration curve showed significant deviation from the ideal diagonal line, especially in the higher range of predicted probabilities. This indicated that, for predicted probabilities between approximately 0.6 and 1, the model tended to overestimate the likelihood of positive outcomes. The higher Brier Score of 0.101 in the test set compared to the training set suggested a decrease in calibration accuracy on unseen data (as shown in Figure 3). The decision curve analysis presents an interesting insight into the Bagging Decision Tree model's performance in both the training and testing sets. In the training set, the net benefit of the model closely tracks the “Treat All” strategy until a threshold probability of approximately 0.6, after which it offers a higher net benefit. The test set exhibits a similar pattern, though the model's net benefit starts declining after a threshold probability of around 0.8 (as shown in Figure 4).

**FIGURE 5:**
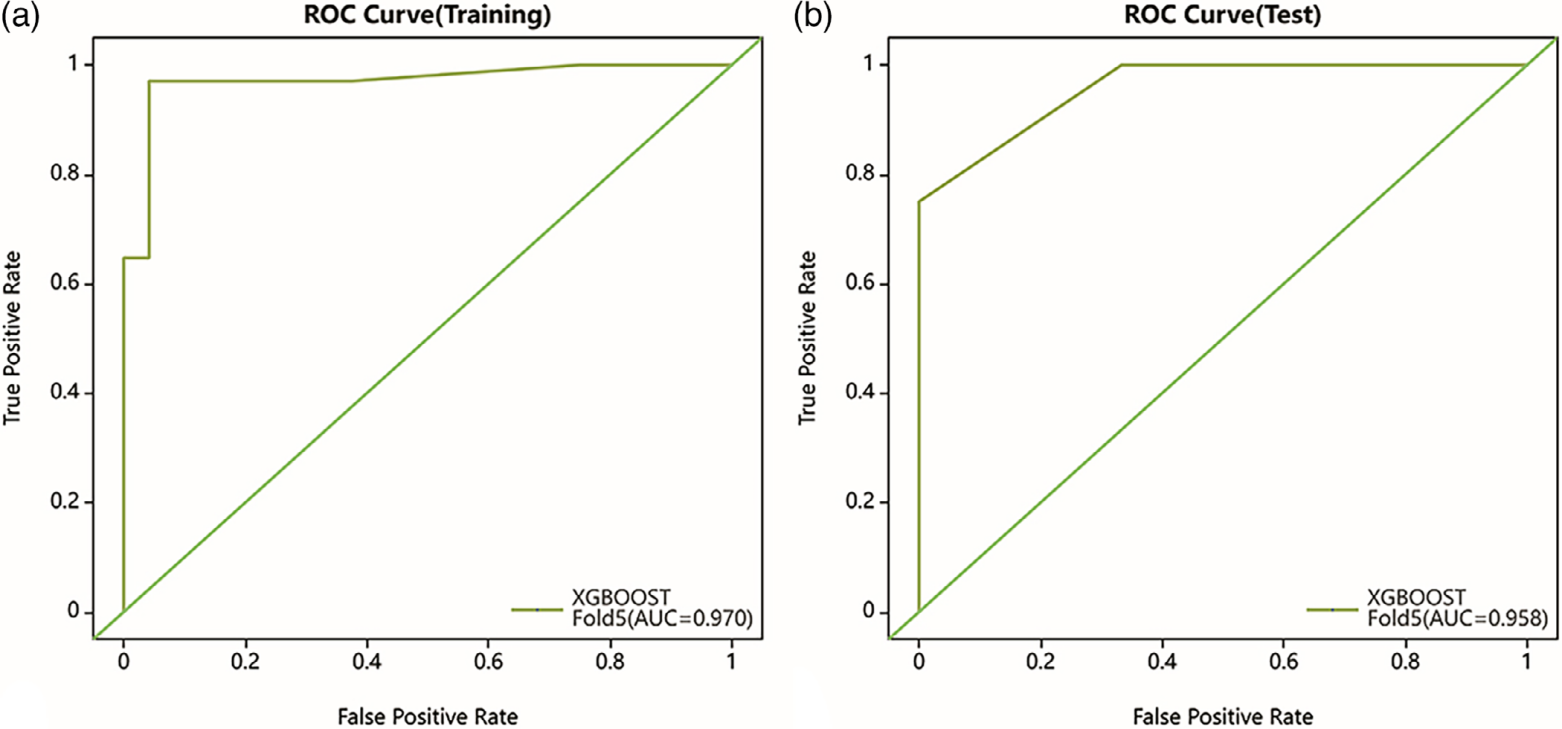
The AUC of XGBoost model.

**FIGURE 6:**
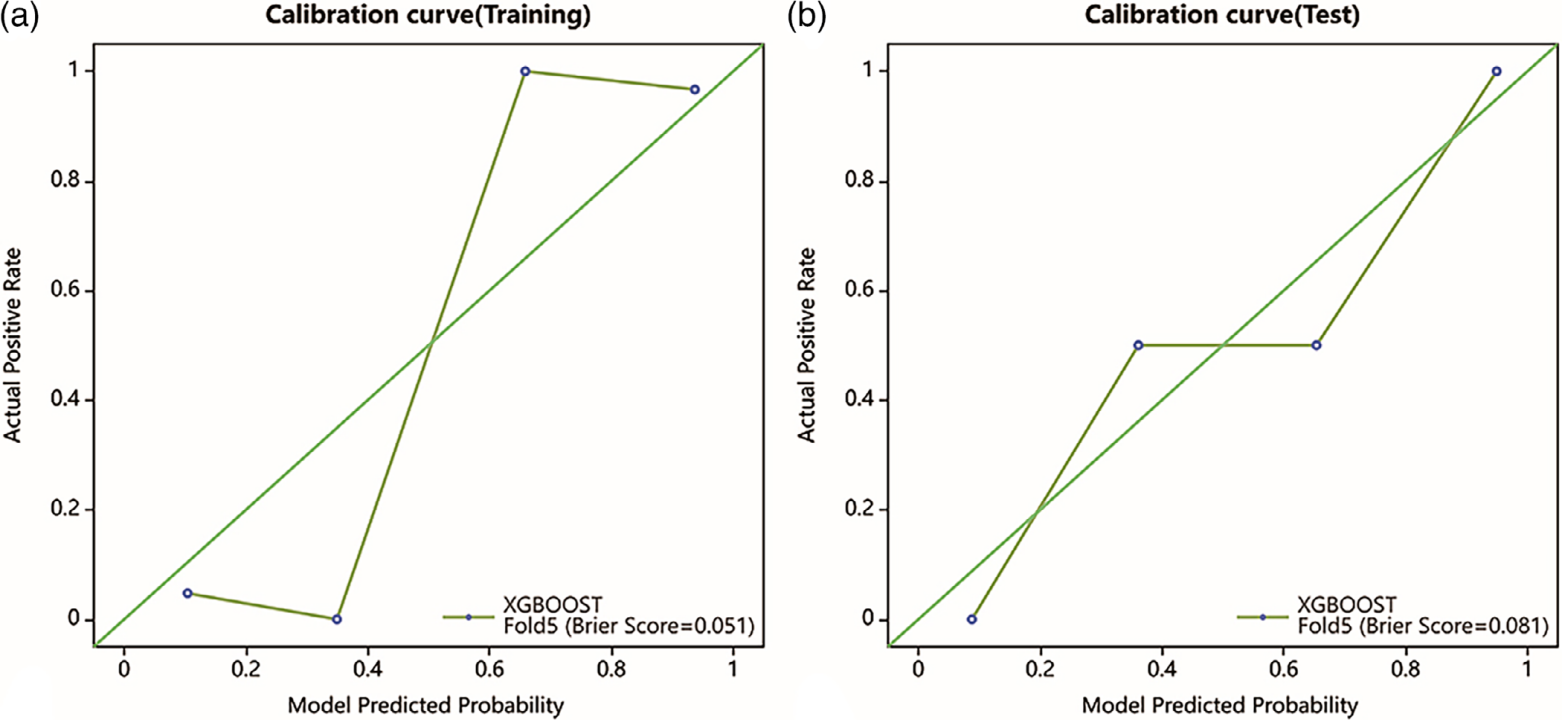
The calibration curve of XGBoost model.

**FIGURE 7:**
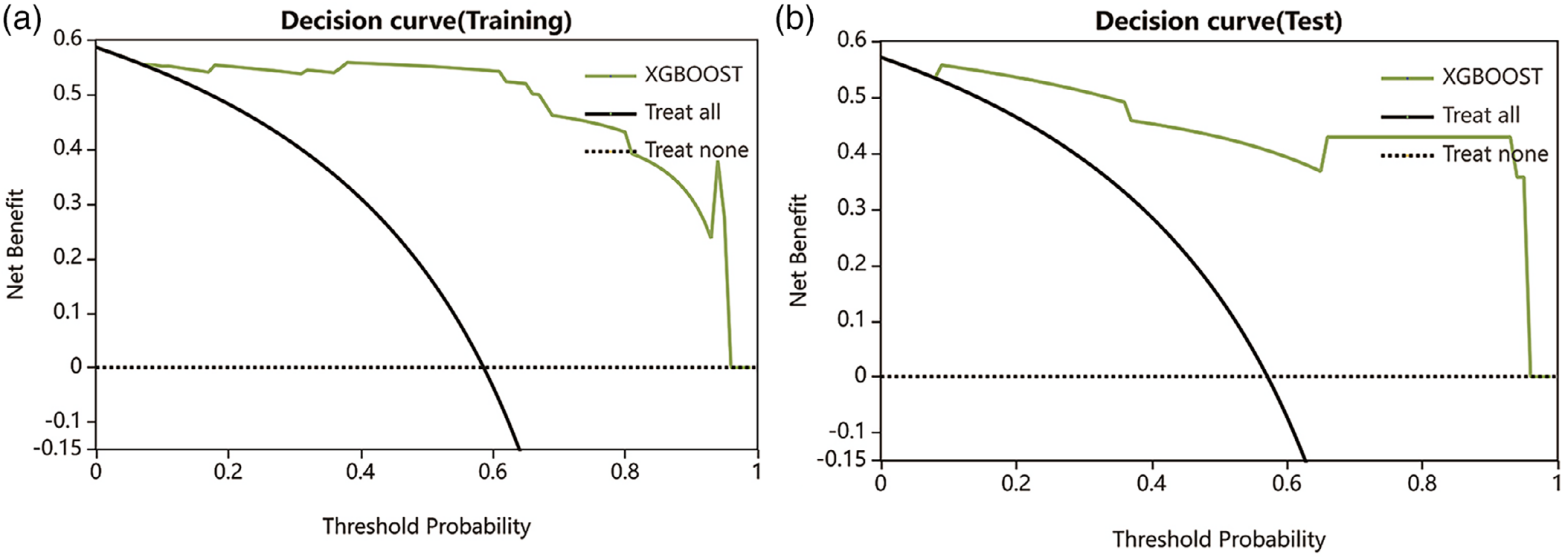
The decision curve analysis of XGBoost model.

Moving to the XGBoost model for osteoblastic lesions, it achieved an AUC of 0.970 (95% CI: 0.927–1) in the training dataset, with a sensitivity of 0.971 and a specificity of 0.875. The model's performance in the test dataset was also robust, with an AUC of 0.958 (95%CI 0.872‐1), along with a sensitivity of 0.875 and specificity of 0.833(as shown in Figure 5).In the calibration curve analysis, the green diagonal line represented perfect calibration, where predicted probabilities aligned perfectly with actual outcomes. However, the curve for the XGBoost model deviated from this perfect calibration, especially around the 0.2 and 0.6 predicted probability marks. This suggested that for certain probability values, the model either overestimated or underestimated the likelihood of positive outcomes.When examining the calibration curve for the test dataset, it was evident that it closely approached the perfect diagonal calibration line, especially when compared to the training dataset. There was a slight deviation at the 0.6 predicted probability mark, indicating some overestimation of positive outcomes. Nevertheless, the overall alignment was better in the test set compared to the training set(as shown in Figure 6). The decision curve analysis, presented in Figure 7, demonstrates the net benefit of using the XGBoost model compared to the two default strategies: “Treat all” and “Treat none”. For the training data, the XGBoost model consistently outperforms the “Treat all” and “Treat none” strategies across a wide range of threshold probabilities. Similarly, in the test data, the model also demonstrates a higher net benefit than both default strategies, especially in the threshold probability range of approximately 0.2 to 0.8.

## 4 DISCUSSION

The ROC curves for osteolytic lesions highlight the excellent performance of the Bagging Decision Tree model on the training set, with a high AUC and a narrow confidence interval. However, the model's performance declined significantly in the test set, with a broader confidence interval extending to 0.66. This discrepancy indicates potential overfitting, possibly due to the relatively high number of features selected by LASSO from a limited dataset.^18^ Future work with larger, multi‐center datasets and more conservative feature selection strategies will be essential to validate and refine these findings.

Furthermore, the observed deviation in the calibration curve at higher predicted probabilities may be explained by several factors. Model overfitting leads to overconfident predictions, and class imbalance might also play a role if positive cases are underrepresented, causing the model to assign excessively high probabilities for certain outcomes. Additionally, the ensemble nature of Bagging Decision Tree—while effective in reducing variance—can sometimes introduce a bias toward overconfident predictions, particularly when the training data do not capture the full variability encountered in real‐world scenarios. The slight miscalibration observed in the test set suggests that the model may have learned some spurious correlations that fail to hold for new samples, especially at higher probability thresholds. This phenomenon may also be related to the diverse imaging manifestations of osteolytic lesions, which could contribute to variability in feature representation and challenge the model's ability to generalize.

In contrast, the XGBoost model exhibits impressive discriminative power, with a high AUC and consistently superior net benefits as shown by the decision curve analysis in both the training and test data. However, given the small sample size and potential overfitting issues, even the XGBoost model may benefit from further refinement. Enhancing the diversity and representativeness of the dataset, applying more rigorous feature selection methods, and incorporating robust regularization techniques could improve calibration and generalizability.To address these issues, future work could explore post‐hoc calibration techniques such as Platt scaling or isotonic regression, as well as strategies like balanced resampling, cost‐sensitive learning, or stronger regularization within the LASSO framework.^19^ Such measures would help mitigate overfitting and ensure that the selected features and model predictions are more robust and clinically applicable.^20^, ^21^This study was designed to evaluate the predictive performance of radiomics features alone to isolate their independent value. One limitation of this study is the absence of an independent external validation dataset, which may limit the generalizability of the model. Although five‐fold cross‐validation was employed to internally evaluate model performance and minimize overfitting, future studies should aim to incorporate independent multicenter datasets for external validation and to enhance the clinical applicability of the model. Additionally, integrating clinical variables in future research may further improve the model's generalizability and real‐world utility.

^89^Sr is a radioactive isotope that emits high‐energy beta‐particles.^22^ Although these particles can penetrate tissues, ^89^Sr is preferentially absorbed by bones due to its chemical similarity to calcium. Metastatic sites, which attract a higher concentration of bone cells, consequently accumulate more ^89^Sr. Once localized, the emitted beta‐particles damage cancer cells, promoting cell death while also alleviating pain and improving bone density and strength.^23^, ^24^ In our study, ^89^Sr therapy achieved an effective rate of 58.33% in osteolytic metastases and 62.16% in osteoblastic metastases. Some osteolytic lesions also showed improvement, suggesting that ^89^Sr may also be beneficial in areas of active bone remodeling, as evidenced by increased MDP uptake on bone scans.^25^

Radiomics has emerged as a transformative approach in medical imaging, enabling the extraction of high‐dimensional quantitative features from standard imaging modalities. It offers several advantages over traditional qualitative assessment. First, it facilitates the precise delineation of anatomical and pathological structures, such as distinguishing tumors from adjacent tissues.^26^ Second, it automates the extraction of texture, shape, and intensity features, enhancing the efficiency and reproducibility of predictive modeling.^27^ Third, radiomics enables quantitative assessment of disease burden and treatment response, providing imaging biomarkers that can forecast outcomes more objectively than clinical impression alone.^28^, ^29^ In the context of therapeutic nuclear medicine, however, the application of radiomics remains relatively limited compared to its widespread use in diagnostic radiology and CT‐based oncology studies.^30^ Traditional evaluation of ^89^Sr treatment efficacy primarily relies on subjective symptomatic relief and post‐treatment imaging, which may lack early predictive power and consistency. In contrast, radiomics offers a noninvasive, pre‐treatment stratification approach that can objectively identify patients more likely to benefit from radionuclide therapy.

This study addresses an important gap in the literature by applying radiomics to predict therapeutic response to ^89^Sr, supporting the development of image‐based decision‐support tools in therapeutic nuclear medicine. Radiomics‐based models have already demonstrated value in other oncologic contexts, such as predicting treatment response in non‐small cell lung cancer^31^ and guiding therapy in head and neck cancer.^32^ Although such tools are not yet widely used in nuclear medicine therapy, this study contributes to expanding their potential for clinical utility in this field. If implemented clinically, such models could help tailor ^89^Sr treatment strategies by identifying patients most likely to benefit, potentially improving overall treatment outcomes and enabling more precise patient management. Further prospective validation and comparison with conventional clinical criteria are warranted to ensure applicability in routine practice.

## 5 CONCLUSION

The integration of advanced modeling techniques with radiomics in medical imaging has the potential to enhance treatment assessment, particularly for ^89^Sr therapy. Although these advancements demonstrate promise, their clinical utility requires further validation. By aiding in the prediction of bone pain relief following ^89^Sr treatment, these tools may provide valuable insights for clinical decision‐making and could serve as a complement to existing approaches.

## AUTHOR CONTRIBUTIONS

Danzhou Fang: drafting of the paper. Yaofeng Xiao and Shunhao Zhou: analysis and interpretation of the data. Feng Shi and Yuwei Xia: conception and design. Gengbiao Yuan and XiaoJiao Xiang: revising it critically for intellectual content. All authors agree to be accountable for all aspects of the work.

## CONFLICT OF INTEREST STATEMENT

The authors declare no conflicts of interest.

## ETHICS STATEMENT

This retrospective study received approval from the institutional review boards of our hospital(No.2023.132) and was performed in accordance with the ethical standards laid down in the 1964 Declaration of Helsinki and all subsequent revisions.

## Supporting information



Supplementary Materials

Supplementary Materials

## Data Availability

The data that support the findings of this study are available from the corresponding author upon reasonable request.
