# Supplementary figures and images for: CT Imaging‐based radiomics predicts the pain relief of Strontium‐89 in treating tumor‐induced bone metastases
Source: J Appl Clin Med Phys. 2025 Jul 15;26(7):e70189. doi: 10.1002/acm2.70189 (PMC12959249; doi:10.1002/acm2.70189)

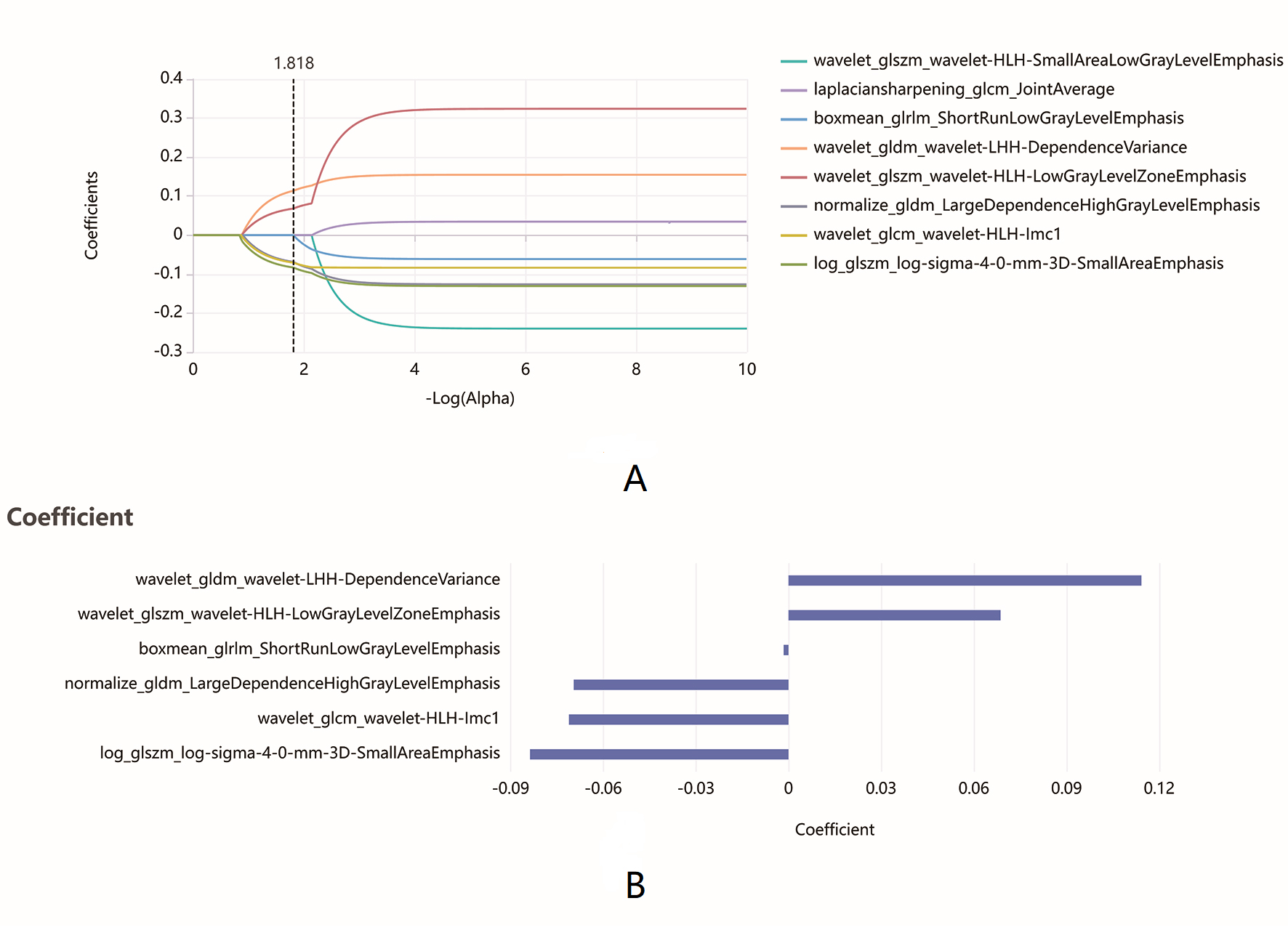

Supplement: Supplementary file 1 — Supplementary Materials [file ACM2-26-e70189-s001.jpg]

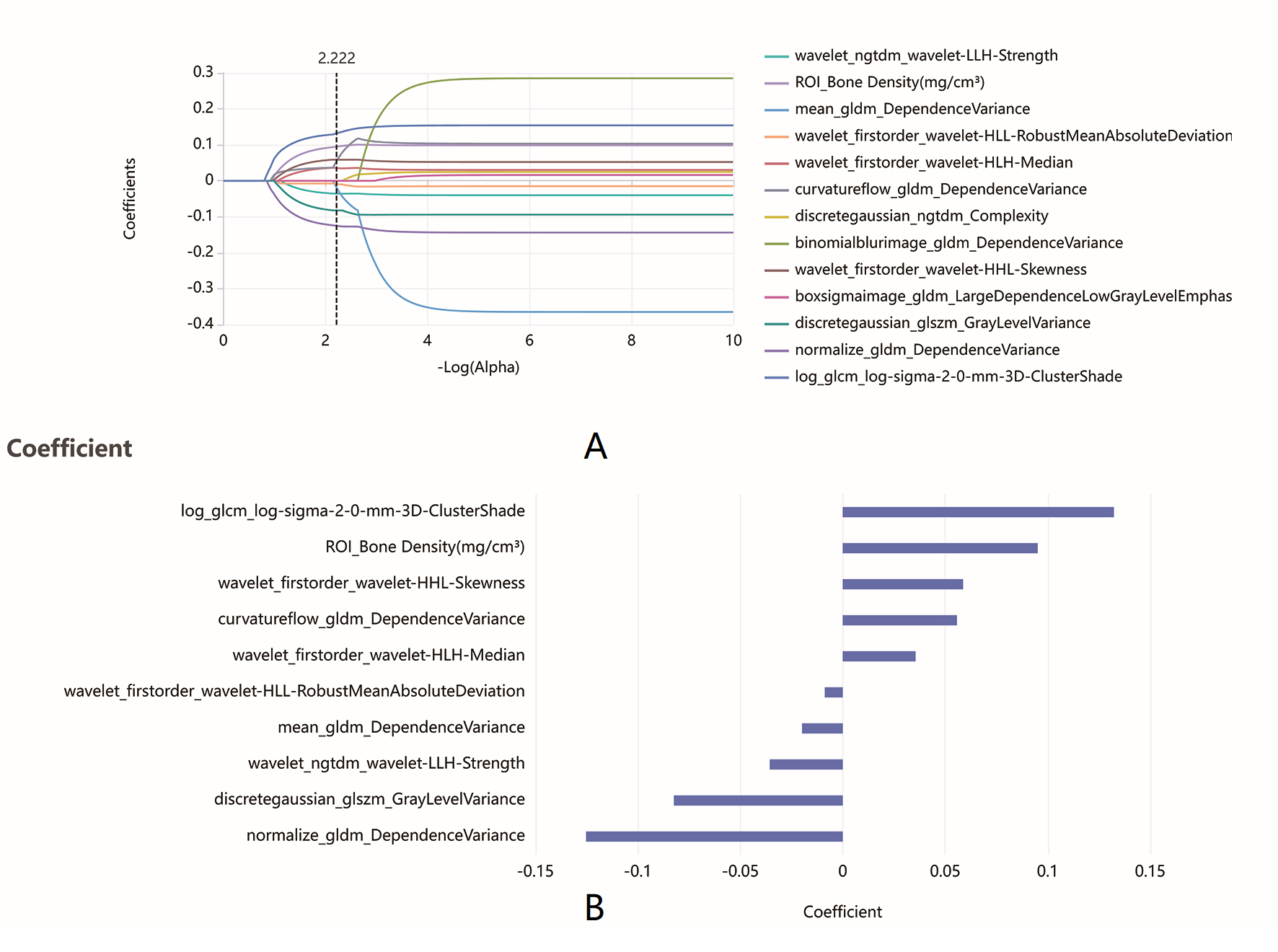

Supplement: Supplementary file 2 — Supplementary Materials [file ACM2-26-e70189-s002.jpg]
